# Papilla preservation periodontal surgery in periodontal reconstruction for deep combined intra-suprabony defects. Retrospective analysis of a registry-based cohort

**DOI:** 10.4317/jced.58265

**Published:** 2021-08-01

**Authors:** Jose A. Moreno-Rodríguez, Antonio J. Ortiz-Ruiz

**Affiliations:** 1 DDS MSc PhD. Private practice, Murcia, Spain; 2 DDS, MSc, PhD. Department of Stomatology, Faculty of Medicine, University of Murcia, Spain

## Abstract

**Background:**

Suprabony defects are the most prevalent defects and there is very little evidence on their treatment. This study aims to assess the effectiveness of papilla preservation periodontal surgery in the periodontal reconstruction of combined deep intra-suprabony defects.

**Material and Methods:**

20 patients with combined intrabony and supra-alveolar deep periodontal defects treated by papilla preservation periodontal surgery were analyzed. Defects were treated with enamel matrix derivate plus xenograft. Clinical recordings made before surgery and at 12 months.

**Results:**

Papilla preservation periodontal surgery showed significant PPD reduction (4.4 ± 1.46 mm; *p*<0.001), clinical attachment gain (3.35 ± 1.6 mm; *p*<0.001), increased REC (1.05 ± 0.94; *p*<0.001), papilla apical displacement (0.85 ± 1.31 mm; *p*<0.005) and KT reduction (0.5 ± 0.76 mm; *p*<0.05). At one week, there was incomplete wound closure and necrosis in 40% and 30% of the treated sites, respectively. At one year, the intrabony component filling was 73.65 ± 27.6 % and the supra-alveolar attachment gain indicated an incomplete intrabony defect resolution (-0.15 ± 1.56 mm).

**Conclusions:**

Periodontal pocket was significantly reduced and the level of clinical attachment increased. However, there was significant recession of the gingival margin and the papilla and a trend to incomplete resolution of the intrabony component.

** Key words:**Periodontitis, surgical flaps, reconstructive surgery, regeneration.

## Introduction

Periodontal surgery is necessary when there are residual and active periodontal pockets of > 5 mm associated with intrabony defects (1). Surgery is indicated to gain access to the deep aspects of the periodontal pocket and to reconstruct the periodontal defects resulting from periodontal disease.

Conventional flap designs in periodontal surgery produce, to a greater or lesser extent, a retraction of marginal tissues during healing, with the formation of soft tissue defects or “gingival craters” in the interproximal aspect associated with soft tissue loss (2). In addition, tissue maturation produces recurrences in the medium to long term, increases the probing depth and a loss of clinical insertion (3).

Microsurgery and new bioactive regenerative agents together with papilla preservation periodontal surgeries (PPPS) have been suggested to produce a minimal wound, minimal flap reflection, and preservation of the papilla (4-7).

Evidence shows that intrabony defects can be treated with good results (6,8,9). Although defects with a supra-alveolar component are more prevalent than intrabony defects10, there is very little evidence about their treatment (11-12).

The objective of this study was to assess the effectiveness of PPPS in periodontal lesions with combined deep intrabony and supra-alveolar defects, assessing the clinical effects of raising the flap and incising the papilla and marginal tissues intrasulcularly.

Material and methods

-Patients.

A retrospective evaluation was carried out in a private clinic in Murcia (Spain) between June 2014 and June 2017. Inclusion criteria were: 1) No relevant systemic condition or disease; 2) Non-smokers or smokers of <10 cigarettes per day; 3) Diagnosis of periodontitis; 4) Full-mouth plaque score < 20%; 5) Non-surgical periodontal treatment and compliance with maintenance therapy for ≥ 1 year; 6) One residual active (bleeding on probing) interproximal pocket combining an intrabony and supra-alveolar defect; 7) Probing pocket depth (PPD) ≥6 mm; 8) Intrabony defect ≥2mm; 9) Defects extending on both buccal and lingual/palatal aspects; 10) Interdental supra-alveolar soft tissue ≥5mm. Exclusion criteria were third molars and teeth with incorrect endodontic or restorative treatment. Twenty patients were included. Each patient provided one interproximal periodontal defect.

All clinical procedures were performed in accordance with the Declaration of Helsinki and Good Clinical Practice Guidelines. The study protocol was approved by the Research Ethics Committee of the University of Murcia (Spain) (protocol number: 2409/2019).

-Clinical parameters

The following measurements were made at baseline and at 12 months using a periodontal probe (PCP UNC 15. Hu-Friedy, Frankfurt, Germany), taking the greatest value as the reference: 1) PPD; 2) Clinical attachment level (CAL); 3) Recession depth (REC); 4) Location of the tip-of-the-papilla (TP) with respect to the cemento-enamel junction (CEJ), with a positive value if coronal to the CEJ or negative if apical to the CEJ; 5) Bleeding on probing (BoP); 6) Keratinized tissue (KT) width.

Intra-surgical clinical measurements were assessed after debridement to determine defect morphology: 1) Distance from the CEJ to the bottom of the defect (CEJ-BD); 2) Distance from the most coronal portion of the bone crest (BC) to the CEJ (BC-CEJ); 3) Intrabony component (INTRA) of the defect defined as the distance from the BC to the BD; 4) 3-wall intrabony component of the defect (3-WALL), defined as the distance from the coronal limit of the three-wall bony component to the BD. (3w-BD); 5) Interdental supra-alveolar soft tissue (SUPRA-ST) by summing the TP and BC-CEJ [the distance from the TP to the BC was not calculated directly as it was not possible to take direct measurements in cases with substantial soft supra-alveolar component without altering the supra-alveolar tissue intrasurgically]; 6) Number of bony walls (3-walls, 2-walls and 1-wall): combination of the number of bony walls (n/n-wall).

Wound closure (WC) at 1 week after surgery 9: 1) complete wound closure (CWC=2), 2) incomplete wound closure (IWC=1) and 3) necrosis (NT=0) of interproximal tissue.

The following values were also calculated at 12 months post-surgery from the parameters recorded: 1) the percentage fill of the intrabony component of the defect, CAL (%) = [CAL gains/INTRA] x 100 6; 2) Supra-alveolar attachment gain (SUPRA–AG), indicating the gain in attachment with respect to the bone crest: SUPRA–AG= [BC–CEJ]–CAL change 12.

-Experimental protocol

All patients received non-surgical periodontal treatment and complied with maintenance therapy for at least one year.

Pre-surgical procedure. One to two weeks before surgery, the area to be regenerated received pre-surgical treatment with micro-ultrasonic tips (After Five® Piezo Scaling, Hu-Friedy, Frankfurt, Germany), only instrumenting the first millimeters of the pocket and all the exposed root surface 13. The surgical phase only proceeded when an excellent tone of the soft tissues overlying the defect was achieved. Patients received 2 grams of amoxicillin (Amoxicilina Normon, Laboratorios Normon SA, Madrid, Spain) one hour before surgery. Post-operative pain and inflammation were controlled with ibuprofen (Ibuprofeno Normon, Laboratorios Normon SA, Madrid, Spain): 600 mg was administered at the beginning of surgery and subsequent doses taken as necessary to control pain.

**Figure 1:**
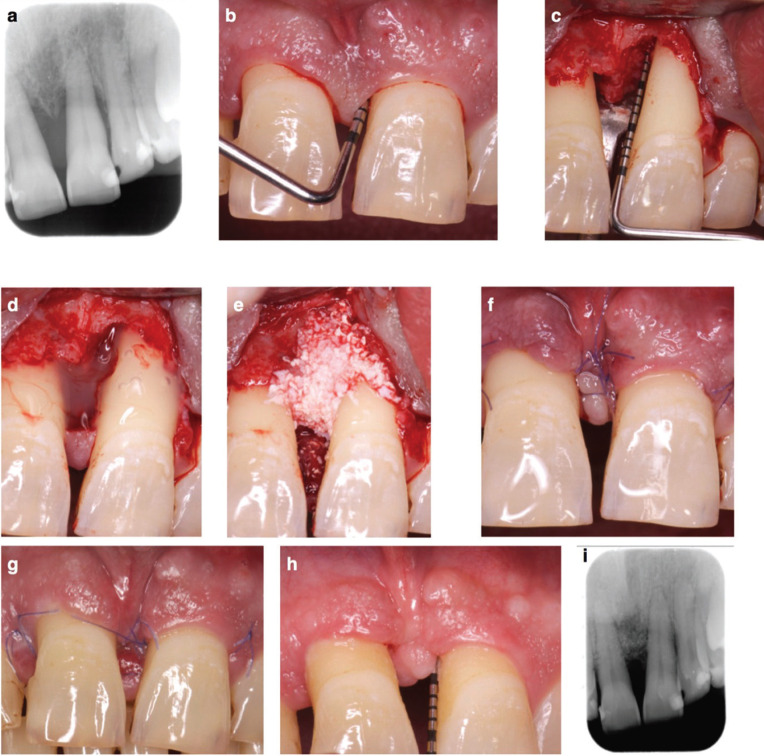
a-b. Baseline periapical x-ray and probing pocket depth; c. Intrasurgical view, combined intrabony plus supra-alveolar type defect; d-e. EMD and xenograft plus EMD mixture application; f. Suture; g. Incomplete wound closure and interproximal tissue partial necrosis at 1 week; h-i. Probing depth and periapical x-ray at 1 year follow up.

Surgical procedures. All surgeries were performed with magnification for improved visual acuity and control. Periodontal defects were assessed by a papilla preservation periodontal surgery (minimally invasive surgical technique, MIST or double flap approach, DFA) (6,8). Interdental tissue was incised using two papilla preservation techniques: the simplified papilla preservation flap (SPPF, interdental space ≤ 2mm)5 and the modified papilla preservation technique (MPPT, interdental space > 2mm)4, based on the width of the interdental space. An intrasulcular incision was extended buccally, lingually and mesiodistally to the two teeth immediately adjacent to the defect. Buccal and palatal/lingual mucoperiosteal flaps were elevated using a papilla elevator and micro-periosteal elevators (Mamadent, Tuttlingen, Germany) until 1-2 mm of the alveolar ridges were exposed (Fig. 1). In very deep defects, the difficult access made it necessary to extend the incision mesiodistally, involving one adjacent interdental space by SPPF o MPPT, in order to elevate the flap without tearing the marginal tissue. Periodontal pocket and granulation tissue were detached with a scalpel micro-blade (Mamadent, Tuttlingen, Germany) and removed with micro-curettes (Micro Mini Five® Gracey, Hu-Friedy, Frankfurt, Germany). Root planing of the fibers attached to cementum was performed with micro-curettes and micro-ultrasonic instruments (After Five® Piezo Scaling tip, Hu-Friedy, Frankfurt, Germany). Biomaterials were then applied and the flap was repositioned and sutured (PGA 6.0, Hu-Friedy, Frankfurt, Germany) by a horizontal internal mattress suture fixing the buccal flap coronal to the mucogingival junction and the base of the lingual/palatal flap, followed by more superficial single sutures adapting both incision borders of the incised papilla to guarantee the primary intention closure (PGA 7.0, Catgut GmbH, Markneukirchen, Germany).

Biomaterial applications. In all treated sites the same biomaterial application protocol was applied. After defect debridement and root surface instrumentation 24% ethylenediaminetetraacetic acid gel (Prefgel®, Straumann, Basle, Switzerland) was applied on the root for 2 minutes. The area was carefully rinsed with saline. EMD (Emdogain®, Straumann, Basle, Switzerland) was applied on the root followed by a composite of deproteinized bovine bone xenograft (Bio-Oss®, Geistlich Pharma, Wolhusen, Switzerland) and EMD (Emdogain®, Straumann, Basel, Switzerland). The composite had to fill only up to the supra-alveolar component of the defect, maintaining the space occupied by the granulation tissue. The composite was placed below a sufficient width of marginal tissue and did not extend to the edges of the incision or hinder its approximation as these must be closed without tension and cured for first intent.

Post-surgical procedures. Patients were instructed to rinse with a 0.2% chlorhexidine digluconate solution (Clorhexidina Lacer, Lacer, Barcelona, Spain) twice a day for 4 weeks. Sutures were removed after 7 days. At 4 weeks, patients were instructed to start brushing with a soft toothbrush and a roll technique. Patients were recalled for control and prophylaxis at weeks 1, 2, 3, and 4 and at 3, 6, and 12 months. The follow up was 12 months.

-Statistical analysis

Patients contributed one defect each. Therefore, the patient was considered as the statistical unit. The sample size (n=20) was calculated a posteriori using CAL values, accepting an alpha risk of 0.05 and a beta risk of 0.20 (power 0.8) in a two-sided test to recognize a difference of ≥ 2 mm as statistically significant and a common standard deviation (SD) of 2. A drop-out rate of 0% was anticipated. (“Sample size and power calculator”, https://www.imim.cat/ofertadeserveis/software-public/granmo/).

Descriptive statistics were used to describe patient characteristics and the specific site, defect morphology, pre-surgical and post-surgical clinical measurements.

Variables were expressed as means and SD. Changes in clinical measurements (baseline and 12 months) were assessed using the student´s t-test for paired data. Cases were categorized according to WC (WC=2 and WC<2). The Student’s t-test or the Mann-Whitney test (according to whether groups were normal or not, respectively) were used to determine the influence of early wound healing on each clinical variable studied. *P*-values < 0.05 were considered statistically significant.

## Results

-Baseline patient and defect characteristics.

Patient characteristics and the defects are shown in Table 1.

**Table 1:**
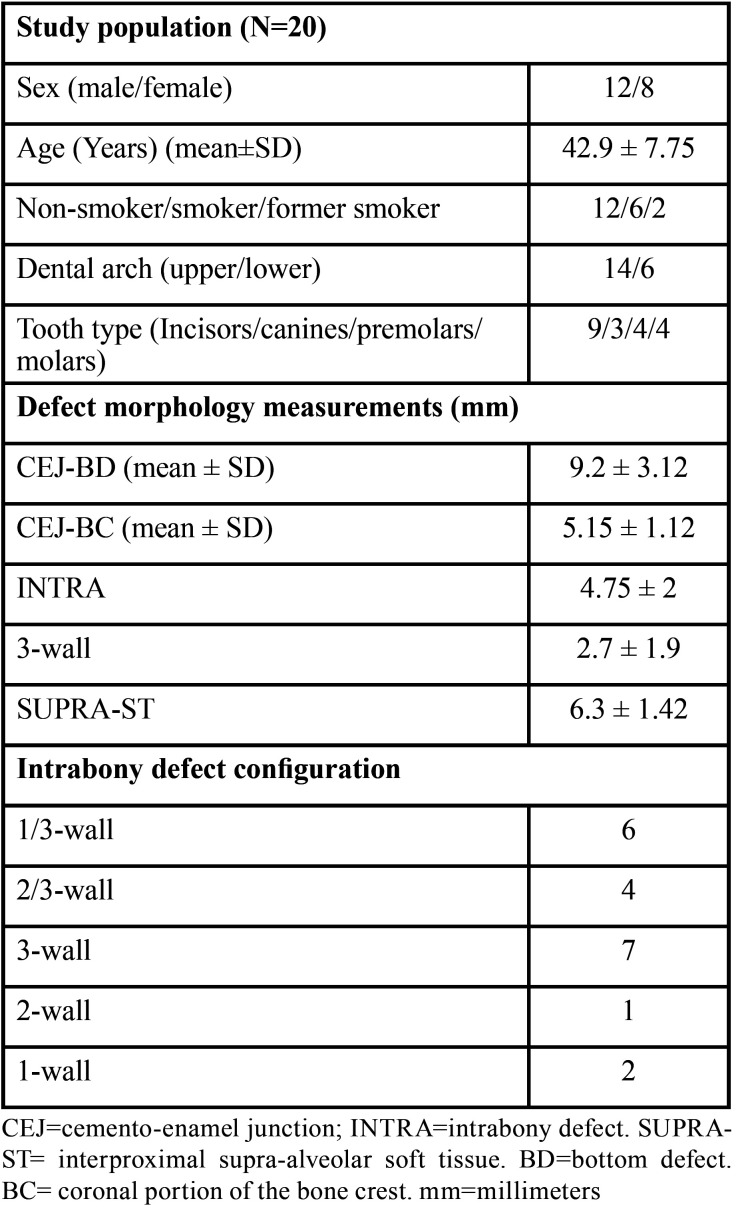
Patient and defect characteristics.

Clinical outcomes at 12 months are shown in Table 2. At baseline, all sites showed a positive BoP, but no site showed BoP at 12 months. PPD and CAL improved significantly from baseline (*p*<.001). However, REC showed a significant increase (*p*<.001). TP showed a significantly apical displacement (*p*=.001) and KT decreased (*p*<.05). After 12 months, the CAL% was 73.65 ± 27.6 % and the mean SUPRA-AG value showed a negative tendency.

Six cases presented CWC, 8 cases IWC and 6 cases NT. One week after surgery, mean WC values were 1.00+/-0.79. Thirty per cent of interventions resulted in primary intention healing (WC=2) (*p*<.001). Significant differences in REC between different WC were found (*p*<0.05). With incomplete flap closure, the mean increase in recession was 1.36+/-0.93 mm and CAL% was 62.98+/-17.41%, while with complete flap closure the mean increase in recession was 0.33+/-0.52 mm and CAL% was 98.61+/-32.67%. No significant differences were observed for other clinical parameters (Table 3).

**Table 2:**
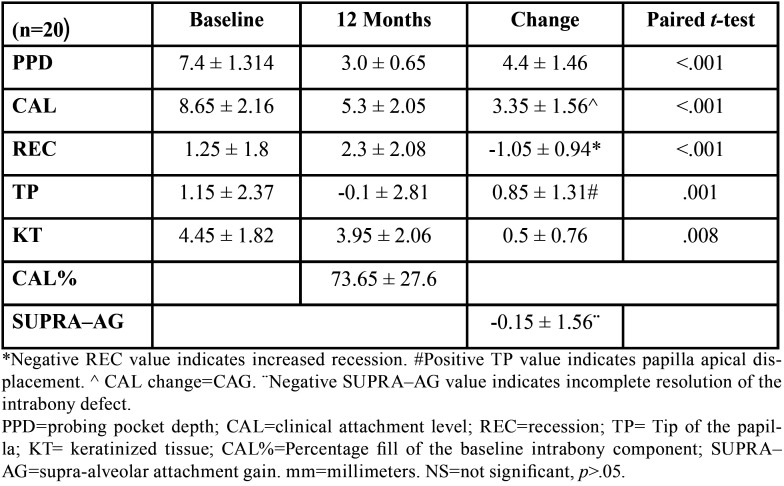
Clinical measurements (mm ± SD).

**Table 3:**
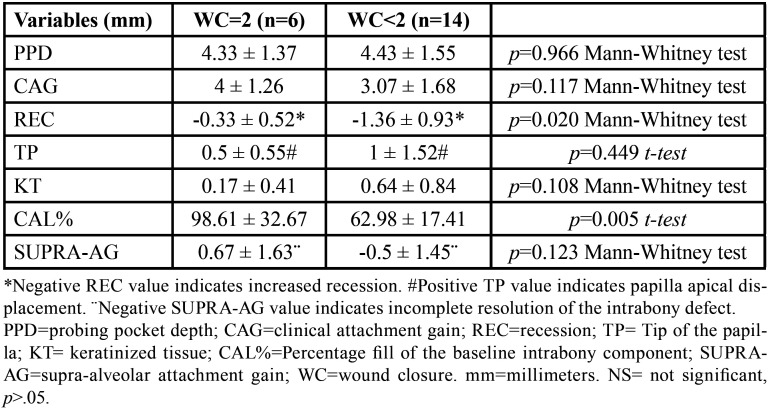
One-year changes in clinical parameters (mm ± SD) in patients with different early wound healing.

## Discussion

Currently, the goal of periodontal surgery is not only to eliminate the periodontal pocket, but to increase clinical periodontal attachment by minimizing postoperative soft tissue contraction. MIST and DFA are similar techniques described by different authors (6,8). The original technique was described for the treatment of pure intrabony defects extending on both buccal and lingual/palatal aspects, limiting the extent of the mesio-distal incision to a minimum. However, in the treatment of deep combined intrabony and supra-alveolar defects, where the alveolar ridge that delimits the defect is located in a deep position, access is difficult and, therefore, it is necessary, in a large number of cases, to extend the incision to one adjacent papilla6, to prevent marginal tissue tearing and facilitate access to the intrabony defect. Extending the incision and widening the flap to adjacent teeth may hinder the stability of marginal tissue in the immediate postoperative period.

There is limited evidence in the treatment of supra-alveolar type defects (11). Furthermore, only one study evaluated the results by PPPS (11). In this study, PPPS significantly improved PPD reduction (PDr) and CAG, as already reported (6,9,14). However, CAG and PDr were associated with a significant increase in REC and TP apical displacement, indicating a tendency to soft tissue contraction. The increase in marginal tissue recession is higher than that obtained in other studies where intrabony defects were treated (6,14) but are similar to other studies where defects with a supra-alveolar component (11) or intrabony defects were treated using PPPS that incised the marginal tissues in order to access the defect (15,16).

Soft tissue healing and the final result of the periodontal reconstruction depends on the vascular damage caused by the incisions (17,18), the capacity of the flap design and the suture techniques to maintain the space for the clot, the wound closure achieved and the stability of the marginal tissues during the healing period (19).

The periodontium and gingival tissue are highly vascularized by an extensive network of supraperiosteal vessels and the plexus of the periodontal ligament, which are connected by intraseptal vessels through the Volkmann canals that cross the alveolar bone (19). The incision at the base of the papilla divides the supraperiosteal vessels above the anastomoses with the periodontal and transseptal plexus; this can severely alter the irrigation above the incision (17-19). Furthermore, the incision in the papilla and subsequent suturing over the defect with limited connective tissue support may compromise the nutrition to the overlaying soft tissue, increasing the risk of wound dehiscence or interproximal soft tissue necrosis during the early healing period (19). After treatment of the defect, the marginal tissues of the flap are approximated and sutured against the surface of the tooth. The avascular nature of the root surface and its hard, smooth and prominent consistency may hinder the stability of the sutured marginal flap, increasing the risk of soft tissue collapse (20). In addition, during the first days of healing, the union of the flap to the dental surface depends almost exclusively on the epithelial adhesion (21), with the force of union of the flap to the root surface being close to 0 N during the first three days and below 6 N during the first and second week (20). In our study, during the early phase of healing, we recorded 40% wound dehiscence and 30% interproximal soft tissue necrosis, results similar to those of other study using PPPS (22). Furthermore, results of this study showed that incomplete wound closure (70%) during early healing significantly increased soft tissue recession compared to complete wound closure (30%). Sutures in place for more than 1 week may induce a subtle pathologic reaction and retard the blood circulation in the papilla near the flap margin (19). In this study sutures were removed after 1 week, furthermore, after this period many of the sutures appear without effective tension.

PPPS are designed to preserve the interproximal soft tissue. However, few studies have evaluated papillary recession (23,24). Our study used TP to assess changes in the location of the papilla with respect to a fixed, sTable reference, the CEJ.

We administered a single dose of antibiotic as a pre-surgical treatment. Although the use of antibiotics for the treatment of periodontal disease is controversial, we administered them because our study included deep residual pockets (≥6 mm) that did not remit with non-surgical periodontal treatment and studies have shown that antibiotic use in the periodontal treatment of residual pockets and/or deep pockets is associated with a greater PDr and CAG (25,26).

RCTs have concluded that, in periodontal intrabony defects treated with a PPPS, the introduction of particulated grafts does not provide a better outcome (8). Our study treated combined intrabony and supra-alveolar defects. Although xenografts do not provide anything positive in the treatment of intrabony defects, its use in supra-alveolar defects may provide the necessary support to the supra-alveolar tissue once the granulation tissue has been removed and would also permit maintenance of the space created and prevent the vertical collapse of the papilla.

We used EMD plus a deproteinized bovine bone xenograft as filling biomaterials for bone defects. The reasons why EMD is used in periodontal regeneration are widely documented (27). However, as they are manufactured or marketed as gels, they cannot prevent the collapse of soft tissue, especially in non-contained defects. Therefore, in these clinical situations, EMD is combined with a bone filler to improve the physical qualities (28).

We assessed the resolution of the intraosseous component by means of two existing indices: the CAL% (9) and the SUPRA-AG (12). As in other study with a papilla preservation flap design (29), the filling component of the intraosseous defect calculated was around 74%, presenting a negative mean SUPRA-AG value, which indicated incomplete resolution of the intraosseous component. A negative SUPRA-AG value also indicates that the periodontal probing remained below the coronal bone peak, conforming a residual intrabony periodontal pocket, which is a risk situation for the persistence of inflammation, recurrence of the periodontal pocket and the loss of alveolar bone (29). In the cases in which complete wound closure during early healing was achieved, there was a significantly higher filling, of around 99%, while in cases where there was incomplete closure, intraosseous filling was 63%. Healing by incomplete closure of the incision line could favor bacterial contamination of biomaterial and prevent clot stabilization, compromising the resolution of the periodontal defect and promoting healing by partial re-epithelialization of the intrabony component with re-establishment of a pocket (21).

Deep combined intrabony and supra-alveolar periodontal lesions can be successfully treated using PPPS and marginal approach, with significant reduction of the periodontal pocket and a gain in clinical attachment, although resulting in significant contraction of the marginal soft tissues, in addition to showing a trend to incomplete resolution of the intrabony component.

In conclusion, wound stability, primary closure and space provision may be difficult to achieve when incising the papilla and raising a flap marginally above the periodontal defect although RCTs are necessary to determine this (9,20). The marginal soft tissue morphology, the interdental space, the avascular nature of the root surface, the confined papillary dimension and the mechanical forces acting on the wound margins may compromise the outcome, increasing supra-alveolar soft tissue contraction (30). Therefore, marginal approaches may provide limited capacity for space provision in non-contained supra-alveolar defects.
